# MCL augmentation using a peroneus longus split tendon autograft satisfactorily restores knee stability with no impairment in foot function and with a low failure rate for concurrent ACL reconstruction

**DOI:** 10.1002/ksa.12522

**Published:** 2024-10-30

**Authors:** Nico Hinz, Maximilian Michael Müller, Lena Eggeling, Tobias Drenck, Stefan Breer, Birgitt Kowald, Karl‐Heinz Frosch, Ralph Akoto

**Affiliations:** ^1^ Department of Trauma Surgery, Orthopaedics and Sports Traumatology BG Klinikum Hamburg Hamburg Germany; ^2^ Department of Trauma and Orthopaedic Surgery University Medical Center Hamburg‐Eppendorf Hamburg Germany; ^3^ Centre for Clinical Research BG Klinikum Hamburg Hamburg Germany; ^4^ Department of Orthopaedic Surgery, Trauma Surgery and Sports Medicine, Cologne Merheim Medical Center University of Witten/Herdecke Cologne Germany

**Keywords:** ACL reconstruction failure, anteromedial instability, MCL augmentation, peroneus longus split tendon autograft

## Abstract

**Purpose:**

Addressing grade 2 and 3 medial‐sided instabilities during anterior cruciate ligament (ACL) reconstruction is crucial to reduce the risk of ACL graft failure. This study introduced a minimally invasive, medial collateral ligament (MCL) augmentation technique using a peroneus longus split tendon autograft, which was fixed to the femoral deep MCL insertion and tibial superficial MCL insertion.

**Methods:**

This prospective, longitudinal, single‐centre case series included patients who underwent MCL augmentation concurrent with primary or revision ACL reconstruction due to anteromedial instability. Preoperatively and at 1‐year follow‐up, clinical examinations, such as rolimeter test of anterior tibial translation and medial instability, knee (International Knee Documentation Committee subjective knee form [IKDC], Lsyholm, Knee Injury and Osteoarthritis Outcome Score [KOOS]) and foot function scores (American Orthopaedic Foot and Ankle Society score [AOFAS]) and complications, were analyzed.

**Results:**

Thirty‐one patients with a mean follow‐up of 13.5 ± 2.6 months and a mean age of 27.8 ± 9.6 years were included. The side‐to‐side difference for anterior tibial translation significantly improved from preoperative to 1‐year follow‐up with an ACL reconstruction failure rate of 6.5%. No patient retained a grade 2 or 3 medial instability on valgus stress testing with 30° flexion. Significant improvements from preoperative to 1‐year postoperative follow‐up were observed in knee function scores: IKDC (48.9 ± 26.9– 71.3 ± 11.5, *p* < 0.001) and Lysholm (59.9 ± 28.5–80.5 ± 11.2, *p* = 0.002) as well as KOOS pain, ADL, sport and QoL, each reaching the respective minimal clinically important difference values. The foot function score AOFAS showed no significant impairment (100 ± 0–99.3 ± 2.5, *p* = 0.250). Complications included cyclops lesions of ACL reconstruction in three patients.

**Conclusion:**

At 1‐year follow‐up, MCL augmentation using a peroneus longus split tendon autograft for patients simultaneously undergoing ACL reconstruction satisfactorily restores knee stability, has a low ACL reconstruction failure rate and does not significantly impair foot function.

**Level of Evidence:**

Level IV therapeutic study; case series.

**Abbreviations**
1‐year FU: 1‐year follow‐up
ACL: anterior cruciate ligament
ADL: activities of daily life
AOFAS: American Orthopaedic Foot and Ankle Society (AOFAS) Ankle‐Hindfoot Score
dMCL: deep medial collateral ligament
IKDC: International Knee Documentation Committee subjective knee form
KOOS: Knee Injury and Osteoarthritis Outcome Score
K‐wire: Kirschner wire
MCID: minimal clinically important difference
MCL: medial collateral ligament (complex)
MRI: magnetic resonance imaging
PCL: posterior cruciate ligament
POL: posterior oblique ligament
pre‐OP: preoperative
QoL: quality of life
sMCL: superficial medial collateral ligament
SSD: side‐to‐side difference
VAS: visual analogue scale

## INTRODUCTION

Combined injuries of the anterior cruciate ligament (ACL) and medial collateral ligament (MCL) are the most frequent multiligament injuries of the knee [38, 60]. While the ACL is the main restraint for anterior translation of the tibia, the superficial MCL (sMCL) is the primary restraint and the deep MCL (dMCL) is the secondary restraint for external tibial rotation [8, 15, 22, 54]. Therefore, a combined injury of ACL and MCL results in an anteromedial rotatory instability [8, 48].

It has been reported that a surgically nonaddressed medial instability grade 2 or 3 results in a 13‐fold increased failure rate for primary ACL reconstruction and a 17‐fold higher failure rate for revision ACL reconstruction [2, 5, 49]. From a biomechanical perspective, an MCL tear causes high strains on the native ACL or an ACL reconstruction [9, 39]. Simultaneous medial reconstruction during ACL reconstruction has been shown to reduce the strain on the ACL reconstruction and restore full knee stability in biomechanical studies [39, 56]. Consistently, an MCL reconstruction in combination with ACL reconstruction lowers the risk of failure for (revision) ACL reconstruction, sufficiently restores knee stability and function and provides good clinical outcomes [3, 18, 27, 35, 58].

Various surgical techniques for MCL reconstruction have been previously described, ranging from anatomical single‐bundle sMCL reconstruction to complex anatomical double‐bundle and triangular sMCL and posterior oblique ligament (POL) reconstruction and nonanatomical MCL augmentation techniques [1, 16, 18, 26, 27, 29, 34, 36, 40, 46, 50, 60]. Alongside the controversial discussion regarding the optimal technique for MCL augmentation to restore knee stability and function, the optimal graft choice for MCL augmentation remains under discussion. In recent years, peroneus longus tendon autografts garnered attention as possible grafts in knee surgery, and have reported favourable functional outcomes and low complication rates for an ACL reconstruction [47, 51, 59]. Therefore, a peroneus longus split tendon autograft could also be of interest for MCL augmentation combined with ACL reconstruction using the quadriceps tendon.

Hence, we developed a minimally invasive, single‐bundle MCL augmentation technique using a peroneus longus split tendon autograft for patients with anteromedial knee instability who simultaneously underwent ACL reconstruction. In addition to describing the surgical technique, we investigated the first clinical outcomes of patients 1 year after simultaneous ACL reconstruction and MCL augmentation using the modified technique. We hypothesized that MCL augmentation using a peroneus longus split tendon autograft will restore knee stability, improve knee function, provide a low failure rate for concurrent ACL reconstruction and does not impair foot function.

## METHODS

### Study design and study cohort

This was a prospective, longitudinal, single‐centre, multisurgeon case study of patients undergoing ACL reconstruction combined with single‐bundle MCL augmentation using a modified technique with a peroneus longus split tendon autograft. Patients with anterior knee instability due to a primary ACL tear or an ACL re‐rupture and concomitant medial instability grade 2 or 3 who underwent simultaneous immediate (within 12 weeks after trauma) or delayed (>12 weeks after trauma) ACL reconstruction and MCL augmentation between December 2020 and May 2022, as recommended by a consensus statement on the posteromedial corner, were included [15]. Patients were included in our outpatient clinic, where the indication for surgical ACL reconstruction and MCL augmentation was established. The indication was based on magnetic resonance imaging (MRI)‐diagnosed ACL and sMCL ruptures (no bony avulsions were present in the study cohort) in combination with clinically determined anterior instability and medial instability grade 2 or 3 in the valgus stress test at 30° knee flexion by two experienced knee surgeons in comparison to the contralateral side. The indication for additional MCL augmentation was verified during examination under anaesthesia in the presence of grade 2 or 3 medial instability on the valgus stress test. Medial instability under valgus stress at 30° knee flexion was classified according to Hughston et al. (grade 1: 0–5 mm; grade 2: 6–10 mm; grade 3: > 10 mm) [5, 24]. Finally, the patients were enroled in this study based on the following inclusion and exclusion criteria:

#### Inclusion criteria

- Patients undergoing MCL augmentation with the below‐described technique using a peroneus longus split tendon autograft concurrent with immediate (within 12 weeks after trauma) or delayed (>12 weeks after trauma) primary or revision ACL reconstruction with quadriceps tendon autograft between December 2020 and Mai 2022 due to anterior instability combined with grade 2 or 3 medial instability with MRI‐diagnosed ACL and MCL ruptures.
- Written informed consent of the patients for participation in our knee ligament register and completion of the 1‐year follow‐up examination.

#### Exclusion criteria

- Patients undergoing ACL reconstruction or MCL augmentation with other techniques as described below in detail.
- Patients with additional ligamentous injuries, for example, of the posterior cruciate ligament (PCL) and/or lateral collateral ligament (except for patients receiving a modified Lemaire procedure due to high‐grade anterior tibial translation and/or for protection of a revision ACL reconstruction).
- Patients with a bony avulsion of the MCL.
- Patients with a history of knee dislocation.
- Patients with an infection of the knee.
- Patients with simultaneous injury of the contralateral leg or multiple traumatological injuries other than knee injury.
- Patients aged <14 years.

At first presentation preoperatively during inclusion and at 1 year postoperatively, clinical examination, including the Lachman test, pivot‐shift‐test, valgus stress test at 30° flexion and side‐to‐side difference (SSD) for anterior tibial translation (Lachman test) using instrumented rolimeter testing, was performed by two experienced knee surgeons compared to the contralateral side. The following scores were collected: Visual analogue scale (VAS) for pain, International Knee Documentation Committee subjective knee form (IKDC), Lysholm score, Knee Injury and Osteoarthritis Outcome Score (KOOS) pain, KOOS symptoms, KOOS activities of daily life (ADL), KOOS sport, KOOS quality of life (QoL) and American Orthopaedic Foot and Ankle Society score (AOFAS). In addition, procedure‐associated complications and any locations of pain were recorded. Procedure‐associated complications with a clearly diagnosable deviation from the expected postoperative course due to the procedure were differentiated from pain alone without a clearly diagnosable cause and were evaluated separately. Concomitant knee injuries and additional surgical procedures were also documented preoperatively.

This was a registry‐embedded study conducted within the framework of the knee ligament register at the BG Klinikum Hamburg. The local Ethics Committee of the Hamburg Medical Association (2020‐10259‐BO‐ff) approved the knee register and the study was performed in accordance with the ethical standards of the 1964 Declaration of Helsinki.

### Surgical technique and postoperative protocol

Surgery was performed after the inflammatory phase, which covers 6 weeks after trauma, and when the knee had an almost free range of motion, it was painless as well as lost swelling. First, knee arthroscopy was performed to detect concomitant injuries, and any meniscal lesions were sutured, or partial meniscectomy was performed for nonrepairable meniscal tears. Single‐bundle ACL reconstruction was performed by an anteromedial approach using a quadriceps tendon graft as previously described [19]. For this technique, an approximately 12 mm wide, partial‐thickness quadriceps tendon autograft without a bone plug, but including a periosteal layer from the patella, was harvested and used as a double‐layered graft. In cases of pronounced anterior tibial translation (SSD > 8 mm) in revision ACL reconstruction, additional extra‐articular lateral tenodesis was performed as described previously [4].

#### MCL augmentation

**Figure 1:**
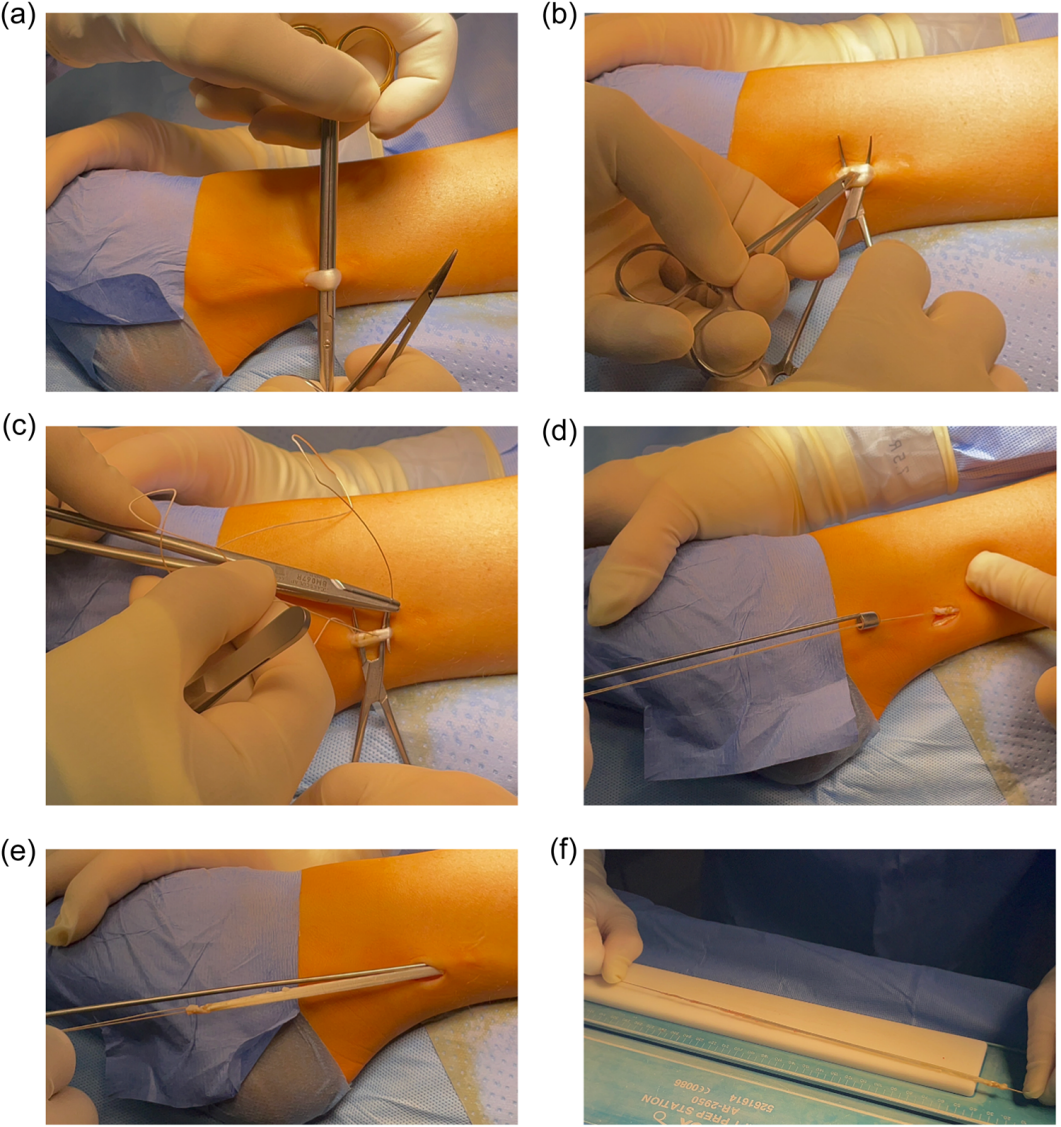
Harvesting the peroneus longus split tendon autograft. First, the peroneus longus tendon is exposed by a lateral incision of approximately 2 cm length located approximately 5 cm cranial to the tip of the distal fibula and at the posterior edge of the fibula directly above the peroneus longus tendon (a). Afterward, the tendon is split into two halves before harvesting (b) and the anterior half is reinforced with a Vicryl suture (c). Finally, the tendon is harvested with a tendon stripper (d and e), and the 15–20 cm long graft is reinforced with another Vicryl suture at the free end (f).

For MCL augmentation, a peroneus longus split tendon autograft was harvested by a lateral incision approximately 2 cm long (Figure 1a). The incision was made approximately 5 cm cranial to the tip of the distal fibula at the posterior edge of the fibula directly above the palpable peroneus longus tendon. This reduces the risk of injury to the sural nerve, which runs more dorsally. The peroneus longus tendon was divided centrally into two halves before harvesting to keep the dorsal half of the tendon intact (Figure 1b). The anterior half was reinforced with a Vicryl suture (#0) and harvested using a tendon stripper (Figure 1c–e). A graft of approximately 15–20 cm in length was obtained (Figure 1f). The tendon graft was doubled, the free end was sutured with a Vicryl suture (#0), and the autograft was deposited in a compress soaked in vancomycin solution until insertion.

**Figure 2:**
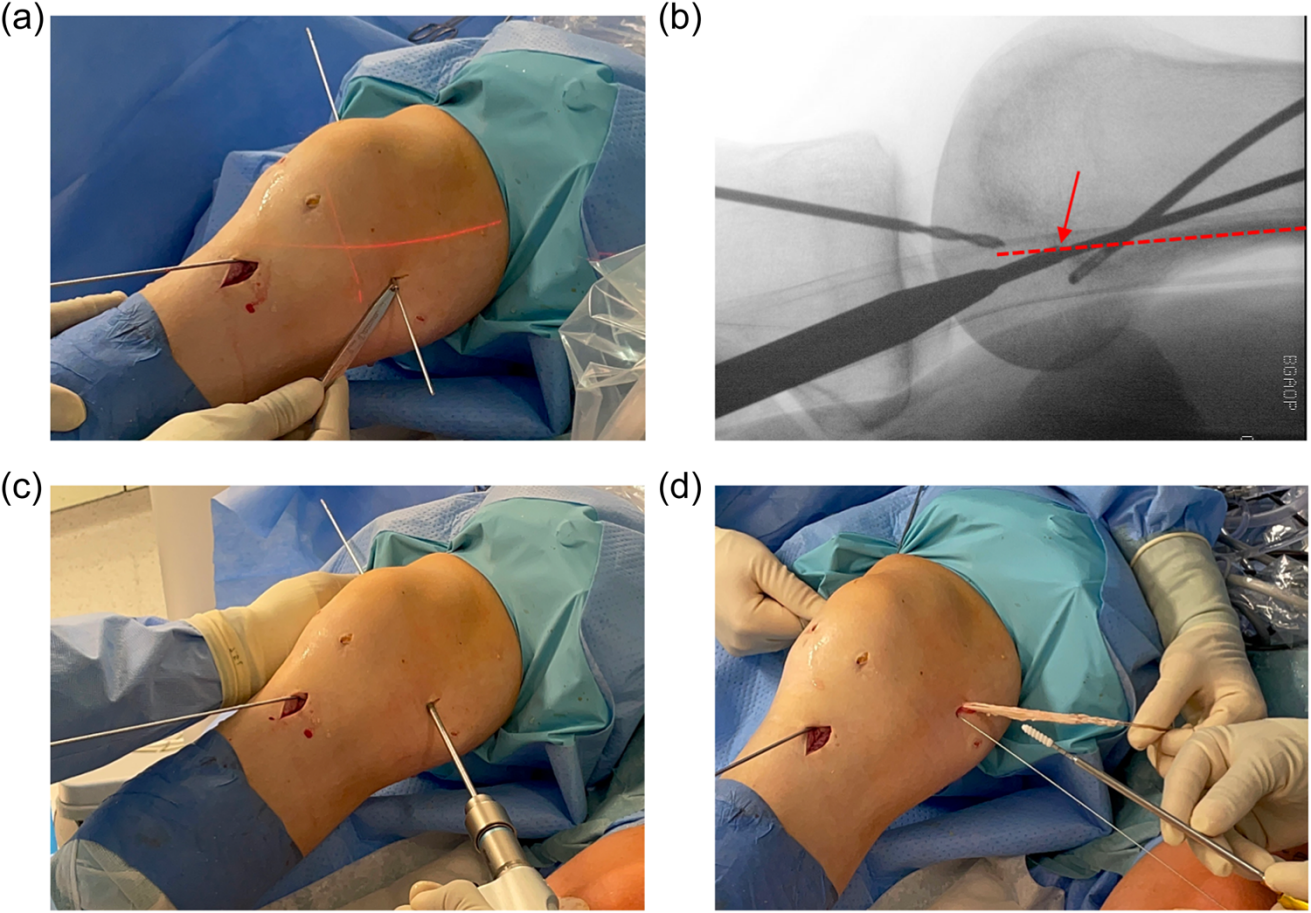
Minimally invasive medial collateral ligament (MCL) augmentation technique. The femoral deep MCL insertion is identified fluoroscopically in a strictly lateral radiation path as the intersection of an imaginary extension of the posterior edge of the femur with the Blumensaat's line and a 2.4 mm K‐wire is inserted. The K‐wires for both anterior cruciate ligament reconstruction and MCL augmentation are shown in the illustrations. A conflict of the drill tunnels can thus be ruled out fluoroscopically and also arthroscopically after overdrilling the wires (a and b). After drilling a 20 mm deep tunnel with a 5–6 mm diameter (c), the graft is pulled doubled in and fixed with an interference screw (d). For a better overview, the femoral insertion of the MCL augmentation was performed before harvesting the quadriceps tendon here, contrary to the usual surgical sequence.

The femoral bone tunnel position was identified fluoroscopically in a strict lateral view. After a 2 cm skin incision and subsequent splitting of the fascia, a 2.4 mm K‐wire was inserted into the intersection of an imaginary extension of the posterior edge of the femur with the Blumensaat's line, which corresponds to the anatomical insertion of the dMCL (Figure 2a,b) [6]. A 20 mm deep tunnel with a diameter of 5–6 mm, depending on the autograft thickness, was drilled via a 1 cm skin incision over the 2.4 mm K‐wire (Figure 2c). The peroneus longus split tendon autograft was pulled and doubled into the femoral bone tunnel and fixed with a 5 × 20 mm interference screw (GENESYS™ Matryx®, Fa. CONMED) (Figure 2d). Subsequently, a 3 cm longitudinal incision was made over the pes anserinus corresponding to the access for harvesting the hamstring tendons, the pes anserinus was exposed and the satorius fascia was split so that the tibial sMCL insertion was displayed. The peroneus longus split tendon autograft was shuttled under the fascia in the direction of tibial sMCL insertion. In the centre of the tibial sMCL insertion, approximately 1–2 cm cranial and dorsal to the pes anserinus and 5–6 cm below the tibial plateau, another 2.4 mm wire was inserted for the tibial tunnel [6, 55]. Before overdrilling the tibial tunnel, the isometry of the MCL augmentation was verified by simulating the fixation of the graft around the K‐wire and testing for no significant length change by flexing the knee. Subsequently, the K‐wire was overdrilled with a 5–6 mm drill bit for a 20 mm deep tunnel. The tibial MCL augmentation tunnel was located dorsally and caudally relative to the tibial ACL reconstruction tunnel. The peroneus longus split tendon autograft was then pulled into the tibial tunnel and fixed with a 5 × 20 mm interference screw (GENESYS™ Matryx®, Fa. CONMED) with the knee in 30° of flexion without any varus/valgus stress and without applying additional tension. Afterward, overtensioning of the MCL augmentation was ruled out by verifying that the posterior horn of the medial meniscus could still be visualized arthroscopically in the inspection of the medial compartment (Figure 2).

The usual surgical sequence involves first harvesting both tendons (quadriceps tendon and peroneus longus split tendon), then inserting K‐wires for ACL reconstruction and MCL augmentation, including radiographic control, and then overdrilling the wires for ACL reconstruction and MCL augmentation so that a tunnel conflict can be ruled out radiographically and arthroscopically. Finally, both grafts were inserted and fixed one after the other.

**Figure 3:**
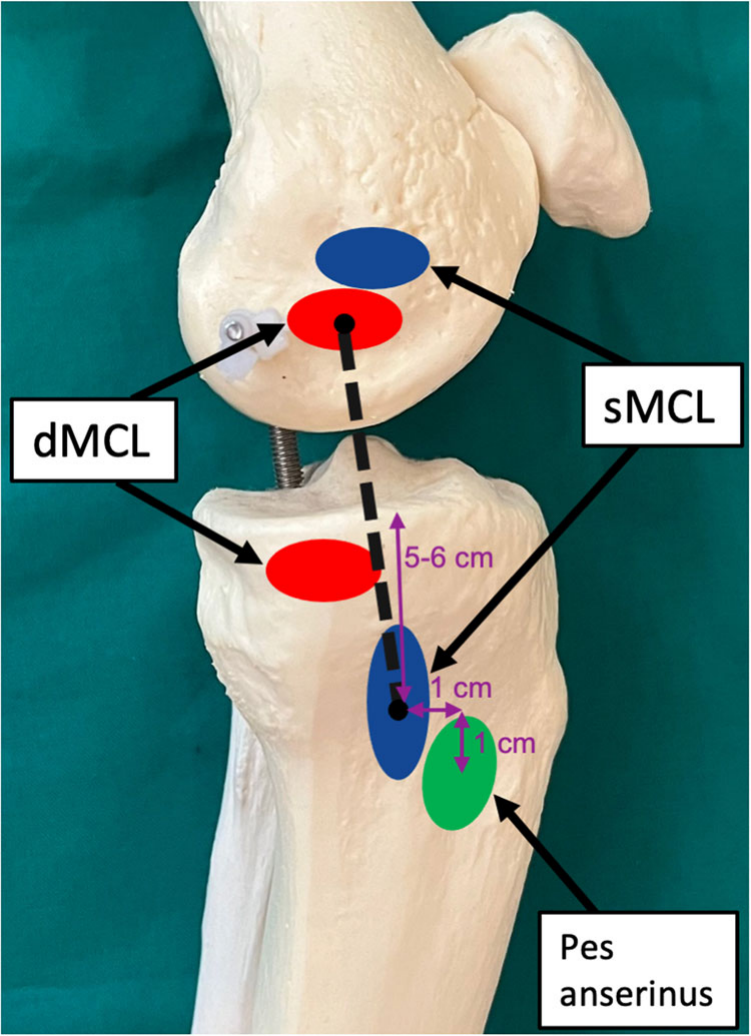
Insertion points (black dots) and course of the medial collateral ligament (MCL) augmentation technique (black dashed line) in relation to the anatomical superficial MCL (blue ovals) and deep MCL (red ovals) insertion points as well as the pes anserinus (green oval). The distances of the tibial insertion point of the graft in relation to the tibial plateau and pes anserinus are displayed in purple.

Consequently, the insertion points of our MCL augmentation technique represent the femoral dMCL insertion and centre of the tibial sMCL insertion, as illustrated in Figure 3 [6, 55].

The patients were mobilized from the first postoperative day with 20 kg partial weight bearing on crutches for 3 weeks, and knee exercising through a physiotherapist was started. The range of motion was not restricted, and no brace was applied. However, valgus stress, anterior tibial translational stress and hyperextension of the knee were not permitted for 6 weeks postoperatively. After 3 weeks, loading was gradually started up to full weight bearing. Isometric exercises and innervation training were recommended in the first 6 weeks after surgery. After 6 weeks, proprioceptive training, coordination training and training with the ergometer were recommended and from the 11th week, running training on level ground was recommended. From the 4th month onward, the focus should have been on muscle building, sports‐specific training, increasing strength endurance and reactive stabilization exercises.

### Outcome parameters

As we aimed to investigate the effect of the new MCL augmentation technique as an additional protective stabilization for ACL reconstruction to reduce the risk of failure, the primary outcome parameters were the failure rate of ACL reconstruction and the change of VAS pain, IKDC form, Lysholm‐Score, KOOS pain, KOOS symptoms, KOOS ADL, KOOS sport and KOOS QoL between preoperatively and 1 year postoperatively. Failure of ACL reconstruction was defined as SSD ≥ 5 mm in rolimeter testing and/or a positive pivot‐shift test grade 2 or 3.

Secondary outcome parameters were the values of rolimeter testing and grade of medial instability (valgus stress test in 30° flexion) in the clinical examination 1 year postoperatively, procedure‐associated complications, any location of pain reported postoperatively and the change in the AOFAS score from preoperatively to 1 year postoperatively. Grades of the pivot‐shift test were as follows: grade 1: glide; grade 2: clunk; grade 3: gross. Medial instability was divided into grades according to Hughston et al.: grade 1: 0–5 mm; grade 2: 6–10 mm; grade 3: >10 mm [25].

### Statistical analysis

Statistical analysis and visualization were performed using GraphPad Prism 9 (GraphPad Software). Concomitant injuries, additional surgical procedures, grade of medial instability, procedure‐associated complications, location of pain and failure rate of ACL reconstruction were expressed as absolute and relative frequencies. Values from the rolimeter test, VAS pain, IKDC Form, Lysholm‐Score, KOOS pain, KOOS symptoms, KOOS ADL, KOOS sport, KOOS QoL and AOFAS were expressed as mean ± standard deviation. Normal distribution was tested using the Shapiro–Wilk test. Longitudinal improvements from preoperatively to 1 year postoperatively were statistically tested using the paired Student's *t* test for parametric data or the Wilcoxon matched pair signed rank test for nonparametric data. Statistical significance was set at *p* < 0.05, and exact *p* values were reported unless *p* < 0.001. The minimal clinically important difference (MCID) was calculated using the distribution‐based method, assuming that half the standard deviation of the preoperative scores for the IKDC, Lysholm, KOOS and AOFAS represented MCID, as described previously [42, 43, 44].

## RESULTS

### Study population

Thirty‐one patients undergoing combined ACL reconstruction with the quadriceps tendon and MCL augmentation with a peroneus longus split tendon autograft due to medial instability grade 2 or 3 between December 2020 and May 2022 were included. Characteristics of the study population are summarized in Table 1. Twenty‐three patients (74.2%) underwent primary ACL reconstruction, whereas eight patients (25.8%) underwent revision ACL reconstruction due to recurrent instability. The detailed characteristics of the patients with primary ACL reconstruction regarding the timing of surgery as well as of the patients with revision ACL reconstruction regarding the indications for revision are shown in Table 1 and trauma mechanisms of the patients with primary ACL reconstruction are listed in Supporting Information S1: Table 1. The concomitant injuries reported were rupture of the medial meniscus in 10 (32.3%), ramp lesion of the medial meniscus in five (16.1%) and rupture of the lateral meniscus in seven patients (22.6%). Consequently, suture/refixation of the medial meniscus was performed in nine (29.0%), partial resection of the medial meniscus in one (3.2%), ramp repair of the medial meniscus in five (16.1%) and suture/refixation of the lateral meniscus in seven patients (22.6%). Additionally, in two patients (6.5%), a lateral extra‐articular tenodesis (modified Lemaire) was performed, both patients with a revision ACL reconstruction. The mean follow‐up time during the study was 13.5 ± 2.6 months.

**Table 1:** Demographic and clinical characteristics of study population.

Characteristics of study population (*n* = 31)	
Sex (*n*/%)	
Male	11/35.5%
Female	20/64.5%
Age (mean ± SD)	27.8 ± 9.6
Type of ACL reconstruction (*n*/%)	
Primary ACL reconstruction	23/74.2%
With immediate reconstruction (within 12 weeks)	9/31.1%
With delayed reconstruction (>12 weeks)	14/60.9%
Mean time from injury to surgery in weeks (mean ± SD)	29.6 ± 38.5
Revision ACL reconstruction	8/25.8%
With traumatic re‐rupture	3/37.5%
With atraumatic recurrent instability	5/62.5%
Concomitant injuries (*n*/%)	
Rupture of medial meniscus	10/32.3%
Ramp lesion of medial meniscus	5/16.1%
Rupture of lateral meniscus	7/22.6%
Additional surgical procedures (*n*/%)	
Suture/refixation of medial meniscus	9/29.0%
Partial resection of medial meniscus	1/3.2%
Ramp repair of medial meniscus	5/16.1%
Suture/refixation of lateral meniscus	7/22.6%
Lateral extraarticular tenodesis (modified Lemaire)	2/6.5%

Abbreviations: ACL, anterior cruciate ligament; SD, standard deviation.

### Clinical examination

**Figure 4:**
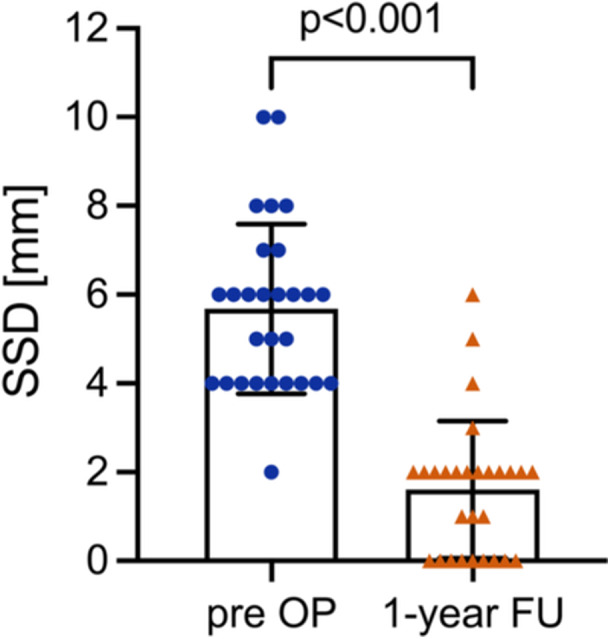
Side‐to‐side difference (SSD) in rolimeter testing preoperatively and at 1 year postoperatively. Columns represent mean, error bars indicate standard deviation and coloured symbols represent individual values. The Wilcoxon test was used for comparison. The exact *p* value is reported if *p* < 0.05.

The SSD in the rolimeter testing of the Lachman test was significantly improved from 5.7 ± 1.9 mm preoperatively to 1.6 ± 1.5 mm in the 1‐year follow‐up (*p *< 0.001) (Figure 4).

ACL reconstruction failed in two patients (6.5%) within 1 year of follow‐up, defined as an SSD of ≥5 mm in rolimeter testing and/or a positive pivot‐shift test grade 2 or 3. One of the patients with ACL reconstruction failure had a primary ACL reconstruction and traumatic re‐rupture due to knee distortion, and the other one had a revision ACL reconstruction and suffered atraumatic recurrent anterior instability. In 12.9% of the patients (four patients), a medial instability grade 1 assessed in valgus stress testing at 30° knee flexion was observed in the clinical examination 1 year postoperatively. None of the patients retained grade 2 or 3 medial instability at the 1‐year follow‐up. The results of the clinical examinations are summarized in Table 2.

**Table 2:** Clinical examination at 1‐year follow‐up.

Clinical examination	
Failure of ACL reconstruction (*n*/%)	2/6.5%
Medial instability (*n*/%)	
Grade 1	4/12.9%
Grade 2 or 3	0

Abbreviation: ACL, anterior cruciate ligament.

### VAS pain, knee function scores and foot function score

**Figure 5:**
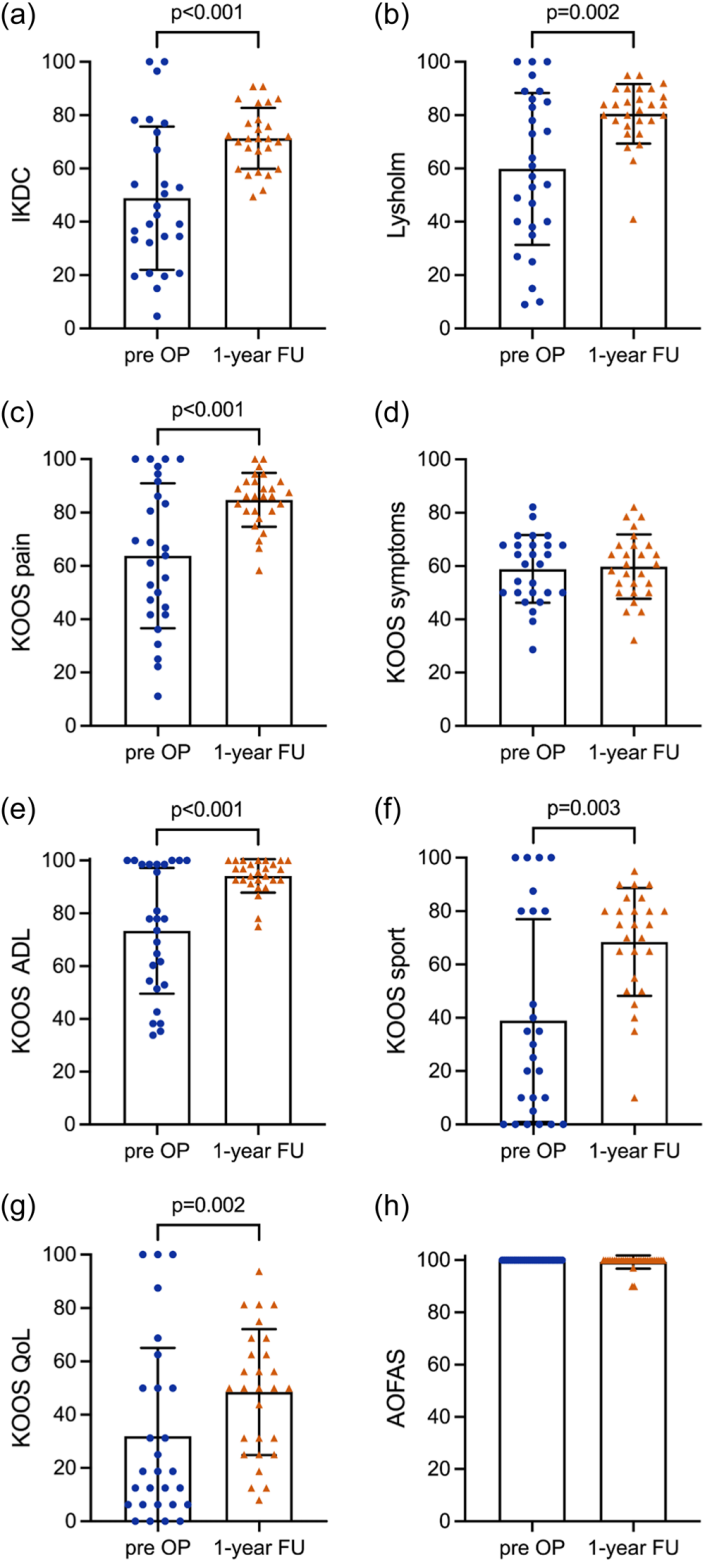
Knee function scores International Knee Documentation Committee subjective knee form (a), Lysholm (b), Knee Injury and Osteoarthritis Outcome Score (KOOS) pain (c), KOOS symptoms (d), KOOS activities of daily life (e), KOOS sport (f) and KOOS quality of life (g), as well as the American Orthopaedic Foot and Ankle Society foot function score (h) preoperatively and at 1 year postoperatively. Columns represent mean, error bars indicate standard deviation and coloured symbols represent individual values. Paired *t* test was used for comparison for (a), (c) and (d), and the Wilcoxon test for (b), (e), (f), (g) and (h). Exact *p* values are reported if *p* < 0.05.

VAS pain significantly decreased from 3.5 ± 2.8 preoperatively to 1.3 ± 1.5 after 1 year postoperatively (*p *< 0.001). The IKDC significantly improved from 48.9 ± 26.9 preoperatively to 71.3 ± 11.5 at the 1‐year follow‐up (*p* < 0.001) (Figure 5a). There was also a significant increase in the Lysholm score from 59.9 ± 28.5 preoperatively to 80.5 ± 11.2 at the 1‐year follow‐up (*p* = 0.002) (Figure 5b). There was no significant difference in the KOOS symptoms between 58.9 ± 12.7 preoperatively and 59.8 ± 12.1 after 1 year (*p* = 0.70). KOOS pain increased significantly from preoperatively to 1 year postoperatively from 63.8 ± 27.2 to 84.7 ± 10.1 (*p* < 0.001), KOOS ADL from 73.4 ± 23.8 to 94.2 ± 6.3 (*p* < 0.001), KOOS sport from 38.9 ± 38.0 to 68.5 ± 20.2 (*p* = 0.003) and KOOS QoL from 31.9 ± 33.2 to 48.5 ± 23.6 (*p* = 0.002) (Figure 5c–g). The mean differences from preoperative to 1 year postoperative were 22.4 in the IKDC, 20.6 in the Lysholm, 21.0 in the KOOS pain, 20.8 in the KOOS ADL, 29.5 in the KOOS sport and 16.6 in the KOOS QoL, reaching all the respective MCID values of 13.4 for the IKDC, 14.3 for the Lysholm, 13.6 for the KOOS pain, 11.9 for the KOOS ADL, 19.0 for the KOOS sport and 16.6 for the KOOS QoL. The AOFAS score for foot function showed no significant change from 100 ± 0 preoperatively to 99.3 ± 2.5 at the 1‐year follow‐up (*p* = 0.250) (Figure 5h). The scores are also summarized in Table 3.

**Table 3:** Outcome scores for knee and foot function preoperatively and at 1 year postoperatively.

Outcome score (mean ± SD)	Preoperatively	1 year postoperatively	*p* Value
Pain			
VAS	3.5 ± 2.8	1.3 ± 1.5	**<0.001**
Knee			
IKDC	48.9 ± 26.9	71.3 ± 11.5	**<0.001**
Lysholm	59.9 ± 28.5	80.5 ± 11.2	**0.002**
KOOS pain	63.8 ± 27.2	84.7 ± 10.1	**<0.001**
KOOS symptoms	58.9 ± 12.7	59.8 ± 12.1	0.70
KOOS ADL	73.4 ± 23.8	94.2 ± 6.3	**<0.001**
KOOS sport	38.9 ± 38.0	68.5 ± 20.2	**0.003**
KOOS QoL	31.9 ± 33.2	48.5 ± 23.6	**0.002**
Foot			
AOFAS	100 ± 0	99.3 ± 2.5	0.250

Abbreviations: ADL, activities of daily life; AOFAS, American Orthopaedic Foot and Ankle Society score; IKDC, International Knee Documentation Committee subjective knee form; KOOS, Knee Injury and Osteoarthritis Outcome Score; QoL, quality of life; VAS, visual analogue scale.

### Pain and procedure‐associated complications

At the 1‐year follow‐up, three patients (9.7%) reported pain at the MCL augmentation site, two patients (6.5%) each showed pain centrally in the knee or anterior knee pain and two patients (6.5%) reported mild pain at the peroneus longus split tendon harvesting site. Regarding procedure‐associated complications, three patients (9.7%) had cyclops lesions of the ACL reconstruction, which were diagnosed using MRI, one of them had primary ACL reconstruction and two of them underwent revision ACL reconstruction. In one (3.2%) of the three patients with cyclops lesions, an additional suture granuloma was reported at the 1‐year follow‐up. Pain and procedure‐associated complications are summarized in Table 4.

**Table 4:** Pain and procedure‐associated complications 1 year postoperatively.

Pain and complications	
Pain at (*n*/%)	
MCL augmentation site	3/9.7%
Centrally in the knee	2/6.5%
Anterior knee	2/6.5%
Peroneus tendon harvesting site	2/6.5%
Complications (*n*/%)	
Cyclops lesion of ACL reconstruction	3/9.7%
Suture granuloma	1/3.2%

Abbreviations: ACL, anterior cruciate ligament; MCL, medial collateral ligament.

## DISCUSSION

The main finding of this study was that the described technique for MCL augmentation with a peroneus longus split tendon graft resulted in an excellent restoration of knee stability, moderate functional outcome and low rate of complications in patients simultaneously undergoing ACL reconstruction. Anterior stability was sufficiently restored, with an ACL reconstruction failure rate of 6.5%. Harvesting the peroneus longus split tendon as a graft for MCL augmentation did not significantly affect foot function.

In the literature, anatomical single‐bundle reconstructions of the sMCL, such as those described by Marx et al., Kitamura et al. and LaPrade et al., are contrasted with nonanatomical MCL reconstruction techniques, such as that of Bosworth/Lind by retaining the tibial attachment of the semitendinosus tendon and fixing the graft at the femoral sMCL insertion [27, 34, 36, 40]. However, the original technique from Bosworth/Lind was modified by now attaching the reconstruction to the distal MCL tibial attachment using suture anchors [33]. Interestingly, a biomechanical study by Kittl et al. demonstrated that the nonanatomical MCL reconstruction of Bosworth/Lind, with the semitendinosus insertion as the tibial attachment and posterior part of the sMCL insertion as the femoral attachment, provides better isometry than the anatomical sMCL reconstruction described by LaPrade et al., with the centre of the distal tibial sMCL insertion as the tibial attachment and 5 mm posterior + 3 mm proximal to the tip of the medial epicondyle as the femoral attachment [28].

The aim of our MCL augmentation was to develop an isometric construct for medial stabilization. Thus, the femoral insertion of the MCL augmentation was positioned at the intersection of an imaginary extension of the posterior femoral margin with the Blumensaat's line on the true lateral fluoroscopic image. This insertion point represents the femoral dMCL insertion according to the anatomical study by Athwal et al. [6]. The tibial insertion was chosen to be just above and posterior to the pes anserinus in the centre of the tibial sMCL insertion approximately 5–6 cm distal to the tibial plateau as described by Athwal et al. and Wijdicks et al. [6, 55]. Before definitive tibial fixation, the isometry of the MCL augmentation was verified clinically. When biomechanically analyzing the isometry of anteromedial reconstructions, Behrendt et al. demonstrated that the isometry of MCL augmentation is primarily affected by femoral attachment but not by tibial attachment, and that the posterior aspect of the femoral sMCL insertion results in the most optimal isometry [11]. The posterior aspect of the femoral sMCL insertion lies almost on the proximal extension of the dMCL insertion used for the isometric MCL augmentation technique described herein, which also supports its isometry.

In addition to a single‐bundle reconstruction of the sMCL, various modifications with additional reconstruction of the POL as a double‐bundle or triangular technique have been described in the literature to better restore the anatomy of the MCL complex [17, 18, 26, 29, 34, 36, 46, 50]. For example, in an in vitro biomechanical study, Coobs reported that their technique of anatomical double‐bundle reconstruction of the sMCL and POL nearly restored normal medial knee stability [16]. LaPrade et al. reported the clinical outcome of this anatomical double‐bundle technique with a mean follow‐up period of 1.5 years and demonstrated an improvement in functional scores and restoration of valgus stability in a case series of 28 patients [34]. However, Zhu et al. demonstrated that single‐bundle sMCL reconstruction is already sufficient to restore anterior tibial translation and external rotation comparable to physiological knee values [60]. Furthermore, a recent biomechanical cadaver study revealed that the POL does not mainly contribute to anteromedial stability, but that the sMCL, especially its anterior fibres, mainly contributes to resisting anterior tibial translation, thereby lowering the strain on ACL reconstruction [23]. Consistently, Behrendt et al. recently demonstrated in a biomechanical study that additional anteromedial reconstruction augments the restoration of anteromedial rotatory stability [10]. Wierer et al. recently described a modified Lind procedure in a technical paper that retains the attachment of the gracilis tendon at the pes anserinus and fixes the construct to the femoral sMCL insertion point as a kind of extraarticular medial tenodesis. By doing so, they obtained an oblique course of the construct supporting its anteromedial stabilization [53].

Most of the popular MCL reconstruction techniques with two or three limbs of the graft, such as those of Bosworth/Lind and LaPrade, are performed by large open approaches. These larger approaches to the medial knee can cause complications, such as knee stiffness, local nerve injuries, wound healing disorders and persistent medial pain [13, 18, 49, 50]. This study aimed to develop a single‐bundle, minimally invasive technique for MCL augmentation with a lower risk of these complications. This is in line with our 1‐year follow‐up data, with only 9.7% of the patients reporting medial pain compared with up to 16.1% in another study using a triangular reconstruction technique [18]. Additionally, no patient experienced stiffness, wound healing disorders or nerve injury postoperatively in our study.

The failure rate of ACL reconstruction was 6.5% at 1 year postoperatively in this study, which is comparable to the failure rate of primary ACL reconstruction in isolated ACL ruptures of approximately 3%–9% and even lower, as reported in comparable studies of combined ACL and MCL reconstructions of approximately 6%–17% [13, 26, 29, 31, 40, 41, 49, 57]. However, when interpreting these results, it must be considered that these are only the results of a 1‐year follow‐up. Furthermore, the comparability of ACL reconstruction failure rates should be treated with caution, as failure was defined differently. While failure of ACL reconstruction was defined in this study as an SSD ≥ 5 mm in rolimeter testing and/or a positive pivot‐shift test grade 2 or 3, some studies only defined failure when a revision ACL reconstruction was required. In addition to the satisfactory restoration of anterior knee stability, the MCL augmentation described here also generated excellent restoration of medial knee stability as assessed under clinical valgus stress testing with 30° knee flexion by two experienced knee surgeons, according to the Hughston classification [25]. The initial medial instability grade 2 or 3, which was the indication for an additional MCL augmentation in our case series, could be sufficiently restored with the modified MCL augmentation technique; no patient retained grade 2 or 3 medial instability, and only 12.9% of the patients exhibited medial instability grade 1 postoperatively.

We also observed a significant improvement in the knee functional scores from preoperatively to 1 year postoperatively, reaching the respective MCID values for nearly all functional scores (IKDC, Lysholm, KOOS pain, KOOS ADL, KOOS sport and KOOS QoL). However, the functional score values of our initial case series with a 1‐year follow‐up period (IKDC: 71.3; Lysholm: 80.5) were lower compared to those of other case series of MCL reconstruction techniques with a follow‐up of usually 2 years (IKDC: 78.2–84.3; Lysholm: 88.1–91) [29, 37, 50]. Nevertheless, the functional outcome of the MCL augmentation technique described here is comparable with the functional outcome observed in the 2‐year follow‐up by Alm et al. for the MCL reconstruction technique described by Preiss et al. (e.g., Lysholm, 82.9), and is still superior to the results of MCL repair (e.g., Lysholm, 75.1) in the study by Alm et al. [3, 46]. The reasons for the low IKDC and Lysholm scores in the cohort described in this study remain unclear. Possible reasons are the short follow‐up period for the patients, with a possible increase in the scores in the further course, and the inclusion of primary and revision ACL reconstructions in this case series, whereas other studies only included primary ACL reconstructions, which are known to achieve higher outcome scores compared to revision ACL reconstructions [21, 40]. Data from the Danish Ligament Register showed that knee function was worse, even after combined MCL and ACL reconstruction than after ACL reconstruction alone. Similar to the present study, knee function after combined ACL and MCL reconstruction was moderate, with good restoration of stability [35].

The graft choice for combined ACL reconstruction and MCL augmentation is challenging. Because the hamstring tendons dynamically contribute to medial stability, harvesting the hamstring tendons in combined injuries of the ACL and MCL was shown to further impair medial stability and is thus not recommended [15, 20, 30, 47]. A few authors recommend the use of allografts, for example, Achilles tendon allografts. However, allografts were shown to have a longer incorporation time, controversially discussed higher failure rate and cause higher costs than autografts [7, 14, 37, 40, 58]. Recently, peroneus longus tendon autografts have been increasingly considered for knee surgery [47, 51, 59]. However, to the best of our knowledge, information on the use of the peroneus longus split tendon as a graft for MCL augmentation is limited. In 2012, Zhao and Huangfu reported the use of a peroneus longus split tendon autograft for ACL, PCL and multiligamentous reconstructions, including reconstruction of the posteromedial complex, and demonstrated sufficient biomechanical properties compared to hamstring tendons, as well as good clinical outcomes in their cohort with heterogeneous indications for the use of this autograft [59]. Abermann et al. reported in their technical paper that flat MCL reconstruction developed by their group can also be performed with a peroneus longus split tendon autograft, as well as with a semitendinosus autograft or allograft but without comparing these graft types [1]. The advantage of the MCL augmentation technique presented here is that relatively short grafts are sufficient for augmentation, making the peroneal graft particularly suitable, which can be slightly thinner and shorter (7.7 vs. 8.0 mm in diameter and 7.3 vs. 7.5 mm in length for quadrupled grafts) [52] or comparable in diameter and size (8.0 and 8.1 mm in diameter and 6.4 and 6.5 mm in length for a four‐strand graft) [12] compared to a hamstring graft. Although two patients reported mild pain at the harvesting site of the peroneus longus split tendon autograft after 1 year, the AOFAS functional foot score showed no significant difference in the global foot function between the preoperative and postoperative evaluations in our initial case series. This suggests that harvesting a peroneus longus split tendon autograft is a safe technique without major restrictions on foot function. This is in line with other studies reporting no major donor‐site complications, no impairment of foot function scores and no significant decrease in peroneus longus strength after harvesting a peroneus longus split tendon autograft [45, 59].

This study has some limitations. Due to the design of this study as an initial case series, there was a lack of a control group. Thus, a direct comparison with other surgical techniques for MCL augmentation was not possible. Furthermore, the biomechanical effect of the modified technique for MCL augmentation and its difference from other MCL augmentation techniques require a biomechanical study setup and were not the focus of this study. Although the anterior knee instability was objectively measured using instrumented rolimeter testing, no stress radiographs were taken for medial instability, as recommended by the in vitro biomechanical study of LaPrade et al. and by the expert consensus statement for posteromedial corner injuries [15, 32]. This is due to the fact that stress radiographs are not included in the routine examination of the knee register used in this study, but the clinical examination of medial instability was performed by two experienced surgeons as described previously [5]. However, this could have led to an underestimation of medial instability and should be considered when interpreting the clinical examination data on medial instability in this study. Additionally, we focused on the valgus stress test in the assessment of medial instability because this test is routinely performed in the context of our knee register. Consequently, new classification systems for anteromedial knee instability, such as the classification system by Wierer et al. additionally using the anteromedial drawer and external dial tests, cannot be applied to this patient cohort [54]. As the study focused on the description of the surgical technique, only clinical outcome data from an initial case series of 31 patients with a mean follow‐up of 13.5 months were used. The short follow‐up time and small sample size limit the conclusiveness of the study regarding the clinical outcome and failure rate of ACL reconstruction and may have a major effect on the functional outcome scores in comparison to other studies of MCL augmentation techniques. Therefore, it is necessary to continue collecting further clinical data, especially with a longer follow‐up period, to obtain reliable clinical data for the MCL augmentation technique described here. Although the small sample size of 31 patients is comparable to that of other case series on MCL augmentation techniques [26, 29, 37, 40, 50, 57], an expansion of the sample size would also lead to more reliable outcome data in further studies. The aim of this study was to represent the indications for MCL augmentation as realistically as possible, as in other studies [34]. This led to a heterogeneous study cohort with immediate and delayed reconstructions, primary and revision ACL reconstructions and different concomitant injuries and surgical procedures. This must be considered when interpreting data from this study and comparing them with other studies.

## CONCLUSION

This study described a new technique for MCL augmentation using a peroneus longus split tendon autograft. The clinical data of this MCL augmentation technique at 1 year postoperatively report that this technique satisfactorily restores knee stability, causes a low failure risk for concurrent ACL reconstruction and has low complication rates. The use of a peroneus longus split tendon autograft for MCL augmentation resulted in no major limitations in foot function. Further data, especially with a longer follow‐up period, are needed to further investigate the significance of this technique, particularly regarding its functional outcomes.

## AUTHOR CONTRIBUTIONS

**Nico Hinz**: Conceptualization; data curation; formal analysis; investigation; methodology; project administration; visualization; writing—original draft preparation and visualization. **Maximilian Michael Müller**: Formal analysis; investigation; visualization; writing—original draft preparation and visualization. **Lena Eggeling**: Investigation; writing—review and editing. **Tobias Drenck**: Investigation; writing—review and editing. **Stefan Breer**: Investigation; writing—review and editing. **Birgitt Kowald**: Data curation; formal analysis; investigation; project administration. **Karl‐Heinz Frosch**: Project administration; supervision; writing—review and editing. **Ralph Akoto**: Conceptualization; investigation; methodology; project administration; supervision; writing—review and editing. All authors read and approved the final manuscript.

## CONFLICT OF INTEREST STATEMENT

The authors declare no conflict of interest.

## ETHICS STATEMENT

This study was approved by the local Ethics Committee of the Hamburg Medical Association (2020‐10259‐BO‐ff) as a registry‐embedded study within the framework of the knee ligament register at the BG Klinikum Hamburg. The study was performed according to the ethical standards in the 1964 Declaration of Helsinki and its later amendments or comparable ethical standards. Written informed consent was prospectively obtained from all participating patients.

## Supporting information

Supporting information.

## Data Availability

The data on which the results of this study are based are available on reasonable request from the corresponding author.
