# Supplementary material for: MCL augmentation using a peroneus longus split tendon autograft satisfactorily restores knee stability with no impairment in foot function and with a low failure rate for concurrent ACL reconstruction
Source: Knee Surg Sports Traumatol Arthrosc. 2024 Oct 30;33(6):2122–35. doi: 10.1002/ksa.12522 (PMC12104778; doi:10.1002/ksa.12522)
Supplement: Supplementary file 1 — Supporting information. [file KSA-33-2122-s001.docx]

| **Type of trauma mechanism (n=23)** | |
| --- | --- |
| Sports injury (mainly soccer) | 12 / 52.2% |
| Fall from a ladder/scaffold | 5 / 21.7% |
| Fall from a bicycle/E-scooter | 2 / 8.7% |
| Direct impact trauma to the knee | 1 / 4.3% |
| Unknown | 3 / 13.0% |

**Supplementary Table 1.** Trauma mechanisms of the patients with primary ACL reconstruction
